# Prevalence of Possible Idiopathic Normal Pressure Hydrocephalus in Sweden: A Population-Based MRI Study in 791 70-Year-Old Participants

**DOI:** 10.1212/WNL.0000000000208037

**Published:** 2023-12-22

**Authors:** Clara Constantinescu, Carsten Wikkelsø, Eric Westman, Doerthe Ziegelitz, Daniel Jaraj, Lina Rydén, Ingmar Skoog, Mats Tullberg

**Affiliations:** From the Hydrocephalus Research Unit (C.C., C.W., D.Z., D.J., M.T.), Institute of Neuroscience and Physiology, Department of Clinical Neuroscience, Sahlgrenska Academy, University of Gothenburg; Division of Clinical Geriatrics (E.W.), Department of Neurobiology, Care Sciences and Society, Center for Alzheimer Research, Karolinska Institute, Stockholm; and Neuropsychiatric Epidemiology Unit (L.R., I.S.), Department of Psychiatry and Neurochemistry, Institute of Neuroscience and Physiology, Sahlgrenska Academy, Center for Ageing and Health (AGECAP) at the University of Gothenburg, Mölndal, Sweden.

## Abstract

**Background and Objectives:**

Very divergent prevalence rates for idiopathic normal pressure hydrocephalus (iNPH) are reported, probably due to differences in study sample selection and diagnostic criteria. This MRI-based study aimed to determine the prevalence of iNPH and iNPH-specific radiologic changes and their association with clinical symptoms in a large, 70-year-old population-based cohort (Gothenburg H70).

**Methods:**

In this cross-sectional study, disturbances in gait and balance, cognition, and urinary continence were assessed using clinical examination and self-report. MRI was evaluated for iNPH-specific imaging markers. iNPH was diagnosed according to International Guidelines (I.G.). Based on radiologic findings, participants were allocated to 1 of 4 groups: (A) Evans index (EI) ≤0.3 (reference), (B) EI >0.3 without other iNPH-typical radiologic findings, (C) radiologically probable iNPH according to I.G., and (D) radiologically *holistically* probable (*h*-probable) iNPH fulfilling radiologic criteria according to I.G. plus highly iNPH-specific changes according to an experienced neuroradiologist.

**Results:**

The Gothenburg H70 Studies include 791 individuals (377 men, 414 women) born in 1944 who underwent brain MRI. The prevalence of iNPH was 1.5% (2.1% for men, 0.96% for women) according to I.G. Ninety participants (11%) had EI >0.3 without other iNPH-typical radiologic findings, 29 (3.7%) fulfilled the I.G. radiologic probable iNPH criteria alone, and 11 (1.4%) were classified as radiologically *h*-probable iNPH. Forty participants (5.1%) had I.G. radiologic features of iNPH (70% men vs 30% women, *p* = 0.005). Gait disturbances were more common in participants with EI >0.3 without other radiologic iNPH features (B) (33%) compared with the reference group (A) (19%) (*p* = 0.006). All clinical symptoms were more common in participants with I.G. radiologic features of iNPH (C + D) than they were in the reference group (A) (*p* < 0.03).

**Discussion:**

The iNPH prevalence of 1.5% among 70-year-olds, which is considerably higher than earlier reported in this age group, suggests that iNPH may be more common than previously assumed. This is supported by the 5.1% total prevalence of imaging signs of iNPH. Ventriculomegaly without other iNPH-typical radiologic findings may be an early sign of developing iNPH in some patients.

## Introduction

Idiopathic normal pressure hydrocephalus (iNPH) is a treatable cause of gait and balance impairment, cognitive decline, and incontinence in older people. Without treatment, patients deteriorate and show increased mortality compared with that of the general population.^1,2^

Reported iNPH prevalence rates among those aged 65 years or older^3-10^ vary widely, from 0.5% to 8.9%, probably because of differing diagnostic criteria. As demonstrated in a previous study, there was a 40.5% difference in estimated prevalence rates when using International or Japanese guidelines diagnostic criteria.^4^ Participant selection methods have also varied widely: some studies have been specifically designed to detect iNPH cases and have used population-based samples,^3-6,8,10-12^ while others have used door-to-door surveys examining the epidemiology of other diseases,^11,13-15^ clinically based materials,^9^ or questionnaires screening for iNPH to select participants.^4^

Furthermore, epidemiologic studies performed outside Japan have used CT,^3,4,11,13-15^ although MRI is preferred for diagnosing iNPH. The prevalence of the typical radiologic features of iNPH, as possible signs of preclinical disease,^5,8^ and their associations with clinical symptoms have yet to be established among older people.

The aim of this study was to determine the prevalence of iNPH and different iNPH-specific radiologic changes and their association with clinical symptoms in a population-based cohort of 70-year-olds, using MRI and detailed clinical assessment.

## Methods

### Study Population

Data were obtained from the population-based Gothenburg H70 Birth Cohort Studies,^16^ which are multidisciplinary epidemiologic studies of older people. This study used a cross-sectional design. In total, 1,667 men and women living in Gothenburg, Sweden, and who were born in 1944 on birth dates ending with 0, 2, 5, or 8, were invited to participate. In total, 1,203 individuals (559 men, 644 women; response rate 72%) agreed to participate in the study and underwent clinical examination in 2014–2016.^16^ Of these, 791 (377 men, 414 women), who were all included in this study, agreed to undergo structural brain MRI during the same period. Based on the clinical examination and MRI results, participants with iNPH were identified. Participants were allocated to 1 of 4 radiologic groups defined by the presence or absence of iNPH-specific radiologic features (described below).

### Standard Protocol Approvals, Registrations, and Patient Consents

The Regional Ethical Review Board in Gothenburg approved the study. Informed consent was obtained from all participants or their relatives if the participant was unable to provide it. The study was performed in accordance with the Declaration of Helsinki.

### Brain Imaging

All participants were scanned on a 3.0-T Philips Achieva system (Philips Medical Systems, Best, the Netherlands) using a protocol including T1, T2, fluid-attenuated inversion recovery (FLAIR), T2*, and diffusion-weighted imaging.^16^ In this study, only T1-weighted and FLAIR images were used for radiologic assessment.

The T1-weighted images were acquired with the following parameters: 1.0-mm isotropic resolution sagittal slices, time to repeat (TR)/time to echo (TE) = 7.2/3.2, field of view (FOV) = 255 × 256 mm^2^, acquisition matrix = 250 × 250 mm, flip angle = 9°.

The FLAIR sequence was acquired with the following parameters: 2.0-mm isotropic resolution sagittal slices, TR/TE = 4,800/280, inversion recovery delay = 1,650 milliseconds, FOV = 250 × 250 mm^2^, acquisition matrix = 250 × 237 mm, flip angle = 90°.

### Radiologic Assessment

The noncommercial, open-source software 3D Slicer (version 4.11.20210226 for Windows)^17^ was used for image visualization and analysis, and the radiologic assessment was performed on T1-weighted and FLAIR images. The T1-weighted volume sequences were reangled by defining a line connecting the anterior and posterior commissures (AC-PC line)^18-20^ and generating transaxial images parallel to the AC-PC line and coronal images perpendicular to the AC-PC-line. The T1 images had a voxel size of 1 × 1 × 1 mm.

The T1 images were assessed according to a visual quantification protocol which included the following imaging markers: Evans Index (EI),^21^ callosal angle (CA), and temporal horn width. EI was defined as the ratio of the largest width of the frontal horns to the largest inner diameter of the skull in the same transaxial slice; a value higher than 0.3 is considered abnormal.^22^ The maximum diameter of the temporal horns was recorded bilaterally on transaxial images, and the average of the bilateral measurements was registered.^23^ The CA was analyzed on coronal T1 images perpendicular to the AC-PC line at the level of the posterior commissure.^19^ The original nonreformatted FLAIR sequence was used to assess the presence of periventricular white matter changes.

### Reliability Testing

To initially test the reliability of the visual assessment protocol, a medical intern (C.C.) and a senior neuroradiologist (D.Z.) individually evaluated the same 20 participants' T1 images according to the protocol. Interrater agreement for radiologic assessment was strong (intraclass correlation coefficient values between 0.77 and 0.99). C.C. re-evaluated 20 participants' scans to test intrarater reliability, which was >0.74 for all variables. Subsequently, C.C. performed an initial screening assessment of all T1 images according to the visual assessment protocol.

D.Z. re-evaluated all T1 images that in the initial assessment had an EI more than 0.3 and assessed the FLAIR images for periventricular white matter changes to determine whether the participants' MRI data fulfilled the diagnostic criteria for radiologically probable iNPH according to International Guidelines (I.G.)^21^ and whether they could also be classified as probable iNPH according to a more holistic radiologic assessment, outlined below. D.Z. made the final decision regarding radiologic diagnosis in all cases.

### Radiologic Definitions

Radiologically probable iNPH was diagnosed according to I.G. with the following modifications: (1) a CA of <90° was used as a cutoff^20^; (2) mean temporal horn width ≥6 mm was defined as temporal horn enlargement^23^; and (3) aqueductal or fourth ventricular flow void was not assessed because no flow-sensitive sagittal T2 sequences were available for analysis. However, if the fourth ventricle was not dilated and the aqueduct's morphology appeared normal on imaging, the aqueduct was considered patent.

Participants were classified as having radiologically holistically probable iNPH, henceforth referred to as radiologically *h*-probable, on the basis of a combined assessment comprising the definition of radiologically probable iNPH according to I.G. plus a feature of disproportionally enlarged subarachnoidal hydrocephalus (DESH)^24^ and the Radscale.^23^ For a systematic appreciation of the contribution of atrophy to the ventricular enlargement, the degree of atrophy was assessed according to the global cortical atrophy scale,^25^ the posterior atrophy scale,^26^ and the medial temporal atrophy scale.^27^ Visual assessment of the brainstem and cerebellum ruled out obvious cases of progressive supranuclear palsy and cerebellar multisystem atrophy. The presence and extent of lacunar and lobar infarctions were noted and related to the ventricular enlargement.

Based on the evaluation of MRI scans, participants were allocated to 1 of 4 groups considered increasingly more specific for iNPH (A–D) as follows: A, EI <0.3 with no ventricular enlargement (reference group); B, EI >0.3 without other iNPH-typical radiologic findings; C, radiologically probable iNPH according to I.G. without fulfilling the radiologic holistic assessment criteria of D^21^; and D, radiologically *h*-probable iNPH that, in addition to the radiologic I.G. criteria, also fulfilled the radiologic criteria for a highly iNPH-specific holistic classification by an experienced neuroradiologist (see below) (Table 1). Each participant was included in only 1 group. Both observers were blinded to all clinical data.

**Table 1:** Definition of Radiologic Groups

Group	Reference (A)	EI >0.3 without other iNPH-typical radiologic findings (B)	Radiologically probable iNPH according to modified I.G. (C)	Radiologically *h*-probable (D)
Definition	No ventriculomegaly: EI ≤0.3	Ventriculomegaly: EI >0.3	Both a and b: a. EI >0.3 b. No discernible CSF flow obstruction And at least one of the following: 1. Enlargement of the temporal horns (≥6 mm) 2. Periventricular signal changes not attributable to microvascular ischemic change or demyelination 3. Callosal angle <90°	Overall holistic evaluation of highly iNPH-specific features by an experienced neuroradiologist combining I.G. radiologic criteria, DESH, Radscale, atrophy grades

Abbreviations: DESH = disproportionally enlarged subarachnoid space hydrocephalus; EI = Evans Index; I.G. = International Guidelines for clinical diagnosis of iNPH; iNPH = idiopathic normal pressure hydrocephalus; radiologically *h*-probable = holistic typical radiologic features of iNPH according to a neuroradiologist.

The reference group is all individuals without ventriculomegaly: EI <0.3 with data on gait/balance, cognition, and incontinence.

### Clinical Assessment and Definition of Clinical Symptoms

Gait and balance disturbance were assessed by a physiotherapist or research nurses. For gait, time to walk 30 m at self-selected walking speed was measured in seconds.^28^ The severity of gait disturbance was rated as nonexistent, slight, or extensive. Gait disturbance was defined as the presence of slight or extensive walking difficulties and/or follows: 30-m walking time >mean 30-m walking speed of all participants +1 SD.

Balance was assessed through the 1-leg static balance test which measured the ability to stand on 1 leg in seconds, for a maximum of 30 seconds.^28^ The best result of 3 tries per leg was registered. Impaired balance was defined as follows: balance test result <the mean balance result of all participants −1 SD.

Cognitive function was evaluated with the Mini-Mental State Examination (MMSE).^29^ The examination was conducted by a psychiatric nurse or medical doctor. Cognitive impairment was defined as an MMSE score <26 and/or an MMSE score <the mean MMSE of all participants −1 SD.^30^

For these 3 measures, we chose a cutoff of 1 SD to minimize the risk of overdiagnosing participants, rather introducing risk of underestimation.

Urinary incontinence was assessed by self-report and defined as present if participants reported at least one of the following: symptoms of urgency leading to involuntary urination occurring often, occasional involuntary urination in clothes, use of medical aids against involuntary leakage of urine, and use of catheter or uridome.

Data on graded walking difficulties were available in 789 participants, 30-m walking time in 763, balance in 728, cognitive function in 785, and urinary incontinence in 787. One participant had no clinical data on gait/balance, cognition, or urinary incontinence and was therefore excluded from the iNPH prevalence calculation.

### Diagnosis of iNPH

iNPH was diagnosed in accordance with the International iNPH Guidelines—as radiologically probable iNPH (radiologic group C or D) together with gait and/or balance disturbance and either cognitive impairment or urinary incontinence or both, as defined above.^21^ Measurement of CSF opening pressure was not practically feasible and was not performed. In addition, it was not possible to rule out other primary reasons for gait disturbance. Even if we used the same method of application of the I.G. as in 2 earlier epidemiologic studies describing probable iNPH,^3,4^ we realize that we have not fully adapted to the I.G. and therefore termed these participants possible iNPH.

Additional data were obtained from the Swedish National Patient Register containing both hospital discharge diagnoses and specialized outpatient care coded according to *ICD10-SE* to exclude secondary iNPH. Exclusion criteria were a history of severe head trauma, meningitis, or subarachnoid hemorrhage within 1 year from inclusion in this study. No participant eligible for iNPH diagnosis fulfilled these criteria; thus, none were excluded.

### Statistical Analysis

Descriptive statistics were used for the epidemiologic data. The prevalence of iNPH was calculated by dividing the number of persons with possible iNPH by the number of study participants. Differences in sex and clinical symptoms between radiologic subgroups and between those fulfilling radiologic and clinical diagnostic criteria for iNPH compared with the reference group were calculated using the Fischer exact test. Differences in EI and MMSE between radiologic subgroups were tested with the Mann-Whitney *U* test. The χ^2^ test was used to calculate interrater and intrarater reliability. All statistical tests were 2-sided, and statistical significance was assumed for *p* values <0.05. Tests were performed using SPSS 28.0 (SPSS Inc., Chicago, IL).

### Data Availability

The data that support the findings of this study are available from the corresponding author on reasonable request.

## Results

### Prevalence of Radiologic Features of iNPH/Hydrocephalic Features

Among all participants, 16% (95% CI 14.0–19.3) had EI >0.3, 11% (95% CI 0.9–13.9) had EI >0.3 without other iNPH-typical radiologic findings, 3.7% (95% CI 2.5–5.3) fulfilled the I.G. radiologic criteria for probable iNPH alone, and 1.4% (95% CI 0.7–2.6) were classified as having radiologically *h*-probable iNPH. In total, 5.1% (95% CI 3.7–6.9) showed I.G. radiologic features of iNPH (group C + D), with a male preponderance (70% men) compared with the reference group. Of the 5.1% with I.G. radiologic features, 7.5% had DESH.

### Clinical Findings in the Different Radiologic Groups

In the group with EI >0.3 without other radiologic findings of iNPH (B), gait and/or balance disturbance was more common than it was in the reference group (A), whereas the 5.1% of participants fulfilling I.G. radiologic iNPH criteria (C and D) had higher frequencies of gait and/or balance disturbance, cognitive impairment, and urinary incontinence compared with the reference group (A) (Table 2).

**Table 2:** Radiologic Findings: Comparisons Between the Reference Group and Different Radiologic Subgroups

	Reference (A) (n = 661)	95% CI	EI >0.3 without other iNPH-typical radiologic findings (B) (n = 90)	95% CI	Radiologically probable iNPH according to I.G. (C) (n = 29)	95% CI	Radiologically *h*-probable (D) (n = 11)	95% CI	Radiologically probable iNPH according to I.G. + radiologically *h*-probable (C + D) (All participants fulfilling radiologic probable iNPH criteria according to I.G.) (n = 40)	95% CI
Men, %	43.9	40.1–47.8	65.6^a^	54.7–75.1	69^b^	49.0–84.0	73	39.3–92.7	70^a^	53.3–82.9
EI, mean (SD)	0.26 (0.027)	0.256–0.260	0.32 (0.02)^a^	0.315–0.323	0.32 (0.018)^a^	0.313–0.326	0.35 (0.026)^a^	0.334–0.369	0.33 (0.025)^a^	0.320–0.336
MMSE, mean (SD)	29 (1)	29–29	29 (2)	29–29	28 (1.8)^b^	28–29	26 (5)^b^	23–29	28 (3)^b^	27–29
Gait and/or balance disturbance, % (n/N)	19.7 (130/660)	16.8–23.0	33.3 (30/90)^b^	24.0–44.1	41.4 (12/29)^b^	24.1–61.0	45.5 (5/11)^c^	18.1–75.4	42.5 (17/40)^b^	27.4–59.0
Cognitive impairment, % (n/N)	10.6 (70/660)	8.4–13.3	10 (9/90)	5.0–18.6	24.1 (7/29)^b^	11.0–43.9	45.5 (5/11)^b^	18.1–75.4	30 (12/40)^a^	17.1–46.7
Urinary incontinence, % (n/N)	26.6 (176/658)	23.4–30.3	20 (18/89)	12.7–30.3	41.4 (12/29)	24.1–61.0	45.5 (5/11)	18.1–75.4	42.5 (17/40)^b^	27.4–59.0

Abbreviations: EI = Evans Index; I.G. = International Guidelines for clinical diagnosis of iNPH; iNPH = idiopathic normal pressure hydrocephalus; MMSE = Mini-Mental State Examination; N = number of persons within each MRI category examined for the corresponding clinical feature of NPH; n = number of persons within each MRI category with the corresponding clinical feature of NPH; radiologically *h*-probable = holistically typical radiologic features of iNPH according to a neuroradiologist.

The reference group is all individuals without ventriculomegaly, that is, EI ≤0.3, with data on gait/balance, cognition, and incontinence.

a *p* < 0.01 vs reference group.

b *p* < 0.05 vs reference group.

c *p* < 0.1 vs reference group.

No differences were seen regarding the frequency of clinical symptoms between the radiologically probable iNPH and radiologically *h*-probable groups (*p* > 0.05 for all clinical symptoms, data not shown).

### Prevalence of Possible iNPH

The prevalence of possible iNPH was 1.5% (C + D) (12 of 790 participants; 95% CI 0.8–2.7), with a male preponderance (67% men vs 33% women) (Table 3). Prevalence in men was 2.1% (8 of 377; 95% CI 1.0–4.3) and in women 0.97% (4 of 413; 95% CI 0.3–2.6).

**Table 3:** Comparison Between Non-iNPH and Possible iNPH

	Non-iNPH (n = 778)	95% CI	Possible iNPH (n = 12)	95% CI
Men, %	47	43.5–50.6	67	35.4–88.7
Age, y, mean (SD)	70.5 (0.26)	70.5–70.6	70.7 (0.19)	70.6–70.8
EI, mean (SD)	0.27 (0.035)	0.265–0.267	0.33 (0.030)^a^	0.308–0.346
MMSE, mean (SD)	29 (1.4)	29–29	26 (4.4)^a^	23–29
Gait and/or balance disturbance, % (n/N)	21.2 (165/778)	18.4–24.3	100 (12/12)^a^	69.9–100.0
Cognitive impairment, % (n/N)	10.9 (85/778)	8.9–13.4	50 (6/12)^a^	25.4–74.6
Urinary incontinence, % (n/N)	25.9 (201/775)	22.9–29.2	83.3 (10/12)^a^	50.9–97.1

Abbreviations: EI = Evans Index; I.G. = International Guidelines for clinical diagnosis of iNPH; iNPH = idiopathic normal pressure hydrocephalus; MMSE = Mini-Mental State Examination; N = number of persons within each MRI category examined for the corresponding clinical feature of NPH; n = number of persons within each MRI category with the corresponding clinical feature of NPH; non-iNPH = those not diagnosed as probable iNPH according to I.G.; possible iNPH = possible iNPH refers to clinical diagnosis of iNPH according to international guidelines; as radiologically probable iNPH together with gait and/or balance disturbance and either cognitive or urinary incontinence or both.

The non-iNPH group is all individuals not diagnosed as having probable iNPH according to International Guidelines with data on gait/balance, cognition, and incontinence.

a *p* < 0.001 vs non-iNPH.

Thirty-three percent (95% CI 11.3–64.6) of the participants with possible iNPH exhibited the full iNPH symptom triad (gait and/or balance disturbance, cognitive impairment, and urinary incontinence).

If defining possible iNPH as radiologic probable iNPH according to I.G. (C + D) plus gait/balance impairment with no other symptoms, the prevalence of possible iNPH was 2% (17 of 790 participants; 95% CI 1.29–3.49) with a male preponderance (71% men vs 29% women).

Two percent of all participants (11 men, 5 women, 95% CI 1.2–3.3) met I.G. radiologic criteria for probable iNPH (C + D) without fulfilling corresponding criteria for clinical symptoms, and 1.5% (9 men, 3 women, 95% CI 0.8–2.7) fulfilled radiologic criteria for probable iNPH (C + D) but had no disturbance of gait and/or balance, cognitive impairment, or urinary incontinence.

No participants had ventricular shunts on MRI.

### Sample Characteristics

In the total sample of 791 individuals, 84% were born in Sweden and 90% in a Nordic country, 52% were women, and 88% had more than mandatory education. When comparing those who participated in the MRI examination (n = 791) and those who did not (n = 412), there was no difference in the proportion of individuals born in Sweden (84.4% vs 84.7%, *p* = 0.933). Other differences have been published previously.^31^ In short, the MRI group had higher education levels, higher scores on MMSE, and less often dementia compared with non-MRI participants.^31^ There were no differences regarding the proportion of women.^31^

## Discussion

In this large population-based study, the prevalence of possible iNPH according to established diagnostic criteria was 1.5% among 70-year-olds: 2.1% in men and 0.97% in women.

Another 3.5% fulfilled the radiologic but not the clinical criteria for iNPH, possibly representing a preclinical or prodromal stage: follow-up on these groups will clarify the predictive value of the different radiologic changes. Ventriculomegaly without other radiologic iNPH features was associated with gait disturbance. Our prevalence estimates are based on imaging, symptoms, and examination findings—criteria that are very similar to those used in clinical practice.

The 1.5% iNPH prevalence we observed is significantly higher than that reported in all previous population-based studies within this age group. A population-based study that used a similar Swedish study cohort but slightly different methodology (CT instead of MRI) but similar radiologic criteria, reported a prevalence of 0.2% in those aged 70–79 years.^3^ As CT entails a lower imaging-resolution than MRI, both temporal horn width and white matter changes were probably less well visualized in the previously mentioned Swedish study, which may be part of the explanation for higher iNPH prevalence reported in our MRI-based study. A prospective, population-based Japanese study of 790 persons aged 61 or 70–72 years found that 1.5% had features of NPH on MRI, but only 0.5% had possible iNPH.^5^ Three (0.38%) 70- to 72-year-old participants had iNPH, a considerably lower prevalence than we report here. That study's methodology differed significantly from ours: the diagnostic criteria were “iNPH features on MRI,” defined as an EI of >0.3 and a disproportional enlargement of the subarachnoid space and narrowing of cortical sulci at the high convexity of the cerebrum (DESH), and at least 1 clinical feature of iNPH. Had we used the *h*-probable radiologic criteria when calculating the prevalence of iNPH together with the I.G. clinical criteria, the prevalence would have been 0.63%, slightly higher than reported in the Japanese study. In adults aged 65–79 years, one study reported a prevalence of 2.1% without specifying the prevalence in different age groups.^4^ However, they selected participants through self-reporting of iNPH-typical clinical symptoms, risking selection bias and subsequent overestimation of prevalence rates. The prevalence of iNPH in a retrospective study including 170 elderly residents aged 65 years or older in a Japanese rural community was found to be 2.5%.^10^ This study used similar criteria as another prospective, population-based Japanese study.^5^ In a recent systematic review and meta-analysis, the prevalence of iNPH was estimated to be 2% of people aged ≥65 years^32^ (the number of 70-year-olds was not specified). We would like to emphasize the fact that our study exclusively examines 70-year-olds, that is, individuals of a single age, in contrast to previous studies^3-5,10,32^ that cover a wider age range. Given that iNPH prevalence rises with age,^3,4^ our results indicate that the prevalence of iNPH in people in their 70s (i.e., ages 70–79 years) is probably even higher than the 1.5% reported here, stressing the notion that our prevalence estimate surpasses estimates of previous reports in this age group. We believe our prevalence estimates are valid and probably more reliable than some earlier ones. The MR-based radiologic assessment used here is superior to CT-based methods, and the clinical examinations were more detailed than in the population-based Swedish study, which used a similar study cohort.^3^ The use of more sensitive methods for detecting iNPH-specific clinical and radiologic features may also explain the higher prevalence rates reported here.

The prevalence estimate reported here and the finding that no participant with iNPH had ventricular shunt on MRI underline the reports on iNPH incidence and incidence of shunt operations for iNPH that conclude that iNPH is probably a largely underdiagnosed and undertreated disorder.^8,9,32-34^

It is important to keep in mind that the group of participants identified here with possible iNPH, and possibly also those with symptoms and an EI >0.3, represent people who should be subjected to a clinical evaluation and other necessary procedures by a neurologist or neurosurgeon including exclusion of other causes for their symptoms and signs to confirm the diagnosis and to assess eligibility for shunt surgery.

In total, 5.1% of all participants fulfilled criteria for radiologically probable iNPH according to I.G., and 3.5% did not show corresponding symptoms fulfilling clinical criteria for probable iNPH. Of these 3.5%, 2% had isolated symptoms not sufficient for a clinical iNPH diagnosis according to I.G. and 1.5% were considered to have asymptomatic iNPH. Two population-based studies have reported lower prevalences (0.96% and 0.47%) of asymptomatic radiologic iNPH.^3,4^ A prospective, population-based Japanese study reported a prevalence of asymptomatic ventricular enlargement with iNPH features on MRI (AVIM) of 1% in participants aged 61 and 70–72 years and suggested that 0.4% of elderly (age >61 years) individuals with normal ventricular size may develop AVIM in ∼8 years.^5^ At the 10-year follow-up, the prevalence of AVIM was 1.4% among those aged 80 years,^8^ and at the 16-year follow-up, 62.5% of cases previously classified as AVIM had developed into iNPH.^35^ Altogether, there is accumulating evidence that radiologic changes indicating iNPH precede clinical symptoms and signs.

Among the participants (5.1%) with radiologically probable iNPH, 27.5% also fulfilled the radiologic criteria for *h*-probable iNPH. Of these, 45.5% fulfilled clinical criteria for iNPH. Thus, the additive radiologic requirements for *h*-probable iNPH such as DESH and absence of atrophy did not increase the identification of typical clinical iNPH. Notably, the radiologically *h*-probable definition would exclude about three-fourth of possible iNPH patients with clinical symptoms, suggesting overly strict criteria. Studies have shown positive outcomes for shunt surgery in patients with ventriculomegaly lacking tight high convexity sulci or cortical atrophy signs.^36-39^ Thus, avoiding these stringent radiologic criteria for radiologically *h*-probable iNPH is recommended. Radiologic iNPH features correlated significantly with all 4 clinical symptoms, but no numerical differences were found between radiologically *h*-probable and radiologically probable iNPH groups, indicating high sensitivity of the less specific I.G. radiologic criteria in identifying patients with iNPH.

Of all study participants, 16% had EI >0.3. This is higher than the 13% reported in 70- to 79-year-olds,^3^ the 6.46% reported in 61-year-olds and 70- to 72-year-olds,^5^ and the 7% reported in ≥65-year-olds.^6^

EI is the most traditional measurement for ventricular enlargement. However, it is not specific for iNPH. EI is sex-dependent,^40^ and an enlarged EI is also seen in various dementias and in normal aging; a recent study reported that 20% of elderly persons have an EI greater than 0.3.^41^ Therefore, new age-dependent and sex-dependent cutoff values have been suggested, and these could differentiate between iNPH and healthy controls with 80% specificity.^40^ However, applying their values to our study material, only 25% of the cases that were classified as possible iNPH would have been classified as having an abnormal EI. Our data show that all clinical symptoms were significantly more common in those we classified as possible iNPH than in those with non-iNPH and that an EI >0.3 alone had a significant relationship with impaired balance and/or gait. This finding strengthens the notion that an increase in ventricular size is an early radiologic manifestation of iNPH development that may lead to impairment of gait and balance, symptoms considered early clinical manifestations of iNPH.^42,43^ Therefore, the proposed new cutoff levels might be too strict when applied to an iNPH population. Instead, an enlarged EI warrants a thorough search for clinical symptoms, even in the absence of other radiologic abnormalities. The current criterion of 0.3 should serve as a screening purpose of identifying a larger proportion of patients who may deserve further evaluation.

In agreement with previous studies,^3,4,6^ we found a higher prevalence of iNPH in men than in women, although the difference was not significant, probably because of the small sample size. In line with results from an earlier cohort in the H70 studies,^3^ of 5.1% of all participants classified as having radiologically probable iNPH, 70% were men compared with 44% in the reference group. The reason for this male predominance is unclear. Sex differences in vascular risk factor profiles may play a role.

Our study has notable strengths, such as a large and representative population, assessment of clinical symptoms and signs, and the fact that all MRI scans with suspected hydrocephalus were evaluated by an experienced neuroradiologist. However, the study has certain limitations, such as the inability to consider all variables and exclusion criteria outlined in the I.G. The study did not confirm a normal opening pressure, which is currently required for a probable diagnosis of iNPH according to I.G.; however, no participant showed radiologic signs of raised intracranial pressure. Notably, the current Japanese iNPH guidelines allow a diagnosis of iNPH without lumbar puncture. Furthermore, aqueductal flow void assessment was not possible because of a lack of available flow-sensitive sagittal T2 sequences. However, on morphologic images, no signs of aqueduct stenosis or other obstruction of CSF pathways was observed. In addition, some participants lacked information on specific clinical variables, and the assessments of gait and urinary incontinence were based on definitions without the possibility of ruling out other potential causes.

Moreover, the MMSE, used for cognitive assessment, has demonstrated limited effectiveness as a cognitive screening instrument for iNPH, given its inability to identify frontal dysfunction characteristic of the condition. Considering that the Montreal Cognitive Assessment (MoCA) is acknowledged for its heightened sensitivity in assessing cognition among individuals with mild cognitive impairment, the utilization of MoCA in our study could therefore have strengthened our study's cognitive evaluation.

In addition, in this study, we did not fully attempt to exclude those who might have had congenital hydrocephalus. However, the evaluation of all MRIs by an experienced neuroradiologist did not show cases with signs of obstructive hydrocephalus or an anatomy of the ventricular system deviant from what is common for iNPH or other obvious signs of congenital hydrocephalus. Furthermore, no participant had a ventricular shunt. These limitations may have resulted in an overestimation of iNPH prevalence. However, if this is the case, they would probably also have limited our ability to establish associations with radiologic findings. We found strong associations both between radiologic features of iNPH and gait and/or balance disturbance, cognitive impairment, and urinary incontinence, and between all clinical symptoms and those diagnosed as possible iNPH, suggesting that our method of identifying possible iNPH is valid. Finally, the low numbers of participants with possible iNPH and radiologic iNPH features introduce a risk of type II error.

The H70 studies aim to invite representative birth cohorts of older adults living in Gothenburg, Sweden. The only exclusion criterion is language difficulties; only participants who can communicate in Swedish are invited, which may hamper representativity. One previous study investigating representativeness among 70-year-olds born 1930 in the H70 studies showed that participants had higher income, less neuropsychiatric and cerebrovascular diseases than same-aged individuals in Gothenburg.^44^ However, education level and the proportion of individuals born in Sweden did not differ.^44^ In addition, when comparing characteristics between the MRI sample and those who did not participate in the MRI examination, those who participated had higher education levels, higher scores on MMSE, and less often dementia.^31^ There were no differences regarding the proportion of women^31^ or individuals born in Sweden. Since iNPH is associated with lower scores on cognitive tests and dementia, the higher rates of nonparticipation among individuals with lower MMSE score and dementia may underestimate the prevalence of iNPH.

In this large population-based study, the prevalence of possible iNPH was 1.5% among 70-year-olds, higher than previously reported in this age group. Another 3.5% showed radiologic features of iNPH without fulfilling clinical criteria. In both groups, male preponderance was evident. The association between EI >0.3 without other radiologic features of iNPH and gait and/or balance disturbance may indicate that ventriculomegaly alone is an early sign of iNPH in some patients. Our data suggest that iNPH may be even more common than previously assumed.

## Appendix Authors

**Table:**

Name	Location	Contribution
Clara Constantinescu, MD	Hydrocephalus Research Unit, Institute of Neuroscience and Physiology, Department of Clinical Neuroscience, Sahlgrenska Academy, University of Gothenburg, Sweden	Drafting/revision of the manuscript for content, including medical writing for content; major role in the acquisition of data; analysis or interpretation of data
Carsten Wikkelsø, MD, PhD	Hydrocephalus Research Unit, Institute of Neuroscience and Physiology, Department of Clinical Neuroscience, Sahlgrenska Academy, University of Gothenburg, Sweden	Drafting/revision of the manuscript for content, including medical writing for content; study concept or design; analysis or interpretation of data
Eric Westman, PhD	Division of Clinical Geriatrics, Department of Neurobiology, Care Sciences and Society, Center for Alzheimer Research, Karolinska Institute, Stockholm, Sweden	Drafting/revision of the manuscript for content, including medical writing for content; major role in the acquisition of data
Doerthe Ziegelitz, MD, PhD	Hydrocephalus Research Unit, Institute of Neuroscience and Physiology, Department of Clinical Neuroscience, Sahlgrenska Academy, University of Gothenburg, Sweden	Drafting/revision of the manuscript for content, including medical writing for content; major role in the acquisition of data; analysis or interpretation of data
Daniel Jaraj, MD, PhD	Hydrocephalus Research Unit, Institute of Neuroscience and Physiology, Department of Clinical Neuroscience, Sahlgrenska Academy, University of Gothenburg, Sweden	Drafting/revision of the manuscript for content, including medical writing for content; analysis or interpretation of data
Lina Rydén, MD, PhD	Neuropsychiatric Epidemiology Unit, Department of Psychiatry and Neurochemistry, Institute of Neuroscience and Physiology, Sahlgrenska Academy, Center for Ageing and Health (AGECAP) at the University of Gothenburg, Mölndal, Sweden	Drafting/revision of the manuscript for content, including medical writing for content; major role in the acquisition of data
Ingmar Skoog, MD, PhD	Neuropsychiatric Epidemiology Unit, Department of Psychiatry and Neurochemistry, Institute of Neuroscience and Physiology, Sahlgrenska Academy, Center for Ageing and Health (AGECAP) at the University of Gothenburg, Mölndal, Sweden	Drafting/revision of the manuscript for content, including medical writing for content; major role in the acquisition of data; study concept or design
Mats Tullberg, MD, PhD	Hydrocephalus Research Unit, Institute of Neuroscience and Physiology, Department of Clinical Neuroscience, Sahlgrenska Academy, University of Gothenburg, Sweden	Drafting/revision of the manuscript for content, including medical writing for content; study concept or design; analysis or interpretation of data

## Glossary

AC-PC: line connecting the anterior and posterior commissures
AVIM: asymptomatic ventricular enlargement with iNPH features on MRI
CA: callosal angle
DESH: disproportionally enlarged subarachnoidal hydrocephalus
DWI: diffusion-weighted imaging
EI: Evans index
FLAIR: fluid-attenuated inversion recovery
FOV: field of view
*ICD10-SE*: Swedish version of *International Classification of Diseases, 10th Revision*
I.G.: International Guidelines
iNPH: idiopathic normal pressure hydrocephalus
MMSE: Mini-Mental State Examination
MoCA: Montreal Cognitive Assessment
TE: time to echo
TR: time to repeat
